# Digital Divide Magnified for Older Veterans Living Off the Grid

**DOI:** 10.1093/geroni/igab046.457

**Published:** 2021-12-17

**Authors:** Kathryn Nearing, Camilla Pimentel, Eileen Dryden, Laura Kernan, Lauren Moo

**Affiliations:** 1 Veterans Health Administration, Aurora, Colorado, United States; 2 VA Bedford Healthcare System, Bedford, Massachusetts, United States; 3 Veterans Health Administration, Bedford, Massachusetts, United States; 4 VA Bedford Health Care System, Bedford, Massachusetts, United States

## Abstract

Compared to urban Veterans, rural Veterans are more likely to be older (55-74), not employed, have less education, more service-related disabilities and unmet healthcare needs. Interviews with a national sample of community-based outpatient clinic providers described highly-rural Veterans who are “off the grid.” These Veterans, by choice and/or circumstance, do not have access to reliable internet, associated devices or knowledge/skills. Providers described the difficulties of connecting with these Veterans even by phone. The healthcare shift to virtual telehealth modalities in response to COVID-19 highlights the digital divide as a social determinant of health. For “off-the-grid” Veterans, past experiences and present-day circumstances converge to perpetuate and exacerbate inequalities in accessing healthcare. Their situation underscores that telehealth is not a panacea for increasing access to care and confronts us with the moral imperative to reach those with whom it may be most difficult to connect to span social, geographic and digital divides.

